# Factors influencing PrEP adoption in sexual health clinics within Ontario’s public health system: a qualitative study using the Consolidated Framework for Implementation Research (CFIR)

**DOI:** 10.3389/fpubh.2026.1760989

**Published:** 2026-04-22

**Authors:** Emma Nagy, Beatriz Alvarado, Carmela Rapino, Oluwatoyosi Kuforiji, Nicholas Cofie, Bradley Stoner, Nancy Dalgarno, Pilar Camargo-Plazas, T. Hugh Guan, Jorge Martinez-Cajas

**Affiliations:** 1 Southeast Public Health, Kingston, ON, Canada; 2 Department of Public Health Sciences, Queen’s University, Kingston, ON, Canada; 3 Office of Professional Development and Educational Scholarship, Queen’s University, Kingston, ON, Canada; 4 School of Nursing, Queen’s University, Kingston, ON, Canada; 5 Division of Infectious Diseases, Department of Medicine, Queen’s University, Kingston, ON, Canada

**Keywords:** HIV, HIV PrEP, HIV prevention, PrEP adoption, public health, sexual health clinics

## Abstract

**Introduction:**

HIV pre-exposure prophylaxis (PrEP) utilization in Ontario is concentrated in two large urban centers, resulting in geographic and structural inequities in access. Little is known about adoption in Public Health Sexual Health Clinics (PHSHCs) or the implementation factors influencing uneven uptake. This study aimed to: (1) assess the extent of PrEP adoption in PHSHCs; (2) identify barriers and facilitators affecting implementation; and (3) explore factors supporting successful and equitable PrEP implementation.

**Methods:**

Semi-structured interviews were conducted with 18 staff and managers across 12 PHSHCs. The Consolidated Framework for Implementation Research (CFIR) guided data collection and analysis, while the Health Equity Implementation Framework (HEIF) was applied to examine structural, organizational, and socio-cultural influences on PrEP access. Clinics were categorized into five adoption categories using the diffusion of innovations model. A content analysis was done to identify common facilitators, barriers, and equity-relevant factors distinguishing clinics at different adoption categories.

**Results:**

PrEP adoption varied widely across clinics. Two were innovators, providing on-site PrEP; three were early adopters with formal referral pathways; four were early majority, relying on informal referrals; and five lacked established services. Facilitators included strong PrEP knowledge and beliefs, inter-organizational partnerships, and supportive organizational cultures. Key barriers were post-COVID-19 service recovery constraints, limited external prescribers, insufficient medication funding, constrained internal capacity, and social individual-level barriers. Differences between advanced and less advanced adopters reflected perceived relative advantage of PrEP within PHSHC mandates, workflow integration complexity, and the presence of a supportive learning climate. Equity-focused analysis highlighted obstacles affecting clients experiencing socioeconomic disadvantage, rurality, stigma, and limited healthcare navigation capacity.

**Conclusion:**

This study highlights that inequities in PrEP access across Ontario PHSHCs are shaped not only by clinic readiness but also by broader structural and organizational conditions. These findings advance understanding of PrEP adoption outside large urban centers, identifying barriers and strategies to expand services and enhance implementation readiness. Using the CFIR and HEIF, we delineated factors to support adoption in early-stage PHSHCs while learning from those with successful integration. Addressing these factors will be critical to expanding equitable access to PrEP and advancing HIV prevention goals.

## Introduction

Canada and Ontario have endorsed global HIV elimination strategies, aiming to reduce the number of new HIV infections by 2026 (1, 2). Yet, in 2023, new HIV diagnoses in Canada and Ontario were 6.1 and 6 per 100,000 population—rates resembling those from the early 2010s (1). The COVID-19 pandemic disrupted prevention programs, and Ontario’s progress now lags behind comparable jurisdictions (2). Ontario has nearly met the UNAIDS 95–95-95 cascade targets, with 89% diagnosed, 86.7% on treatment, and 97.8% of those virologically suppressed (3). Still, pre-exposure prophylaxis (PrEP)—a proven, highly effective HIV prevention tool—remains underused (4–7). Since the 2017 Canadian PrEP guidelines recommended its use for individuals at substantial risk (identified through HIV risk assessment tools that consider sexual risk exposures, substance use, and prior sexually transmitted infections) (8), PrEP use in Ontario rose 2.3-fold from 2018 to 2022, with 14,650 users in 2022—primarily men (97%) aged 20–39 (59%) with private insurance (76%) (3, 9). The diversity and number of PrEP prescribers have also increased in recent years, with sexual health clinics and online providers expanding PrEP delivery (10). However, overall access to PrEP in Ontario remains lower than in several other Canadian provinces, in part due to differences in funding models (11). In contrast to provinces such as British Columbia and Saskatchewan, where PrEP is publicly funded, PrEP coverage in Ontario is only partial. Full coverage is limited to specific groups, including individuals under the Ontario Disability Support Program, those younger than 25 years, and those aged 65 years and older. As a result, access remains uneven: approximately 75% of PrEP dispensations occur in Toronto or Ottawa, with substantially lower uptake in rural and suburban areas, with a limited number of family physicians aware and interested in its prescription (9, 12).

HIV continues to disproportionately affect populations in Ontario that have historically experienced higher exposure and vulnerability to institutional, community, and interpersonal stigma and discrimination, social exclusion, and poverty. In 2023, 83.3% of new HIV diagnoses in Ontario occurred in gay, bisexual, and other men who have sex with men (GBM 56.8%), African, Caribbean, and Black (ACB) communities (29.8%), women (26.9%), people who inject drugs (PWID; 10.6%), and Indigenous peoples (4.6%) (13). Despite this elevated risk of HIV acquisition, evidence indicates persistently low awareness, uptake, and adherence to PrEP among ACB, cis-gender women, Indigenous, and other racialized communities (14, 15). For example, studies by Adam et al. and Palma et al. found that GBM from racialized communities—including Indigenous, Black Caribbean, and newcomer populations—were less likely to have access to PrEP-prescribing providers and were consequently less likely to engage across the PrEP care cascade (12, 16). Similarly, Ajiboye et al., in a cross-sectional study including individuals of diverse gender identities and sexual orientations, reported that Indigenous participants and those living in rural areas were significantly less likely to be aware of or to use PrEP (17). Van Uum et al., and Orser et al. have identified low PrEP awareness about PrEP in community and clinical samples of women at high risk of acquiring HIV infection (14, 15). These data together with access gaps, particularly outside major urban centers, underscore the urgent need for more equitable, community-informed strategies to expand PrEP availability in urban areas outside major metropolitan cities and rural Ontario.

### The role of public health sexual health clinics

Previous history of sexually transmitted and blood borne infections (STBBIs) are known risk factors for HIV and are included in many national guidelines as clinical indicators for offering PrEP (8). As common points of care for STBBI testing and treatment, public health sexual health clinics (PHSHC) are well positioned to support PrEP scale-up. Their well-established focus on comprehensive sexual health, access to populations at increased risk, and preventive care expertise make them ideal settings for PrEP delivery (10, 18–20). Evidence suggests that integrating PrEP into PHSHC enhances outreach, reduces stigma, and improves coordination of care for those interested in lowering their HIV risk (20, 21). Nurse-led models and telehealth options within PHSHC have also proven feasible, acceptable, and potentially impactful in reducing HIV incidence (10, 18, 22).

Despite this potential, PrEP implementation in PHSHC in Ontario remains limited. As of February 6, 2025, only 7 of Ontario’s 34 public health units listed PrEP services on their websites—mostly limited to referrals. In Southeast Ontario, only one health unit clinic offers on-site PrEP (23). No published studies to date have examined what enables or hinders PrEP adoption within Ontario PHSHC. This study addresses this gap using the Consolidated Framework for Implementation Research (CFIR) (24) and the Health Equity Implementation Framework (HEIF) (25) to explore the following research questions:

1. To what extent has pre-exposure prophylaxis been adopted in PHSHC in Ontario?
2. What barriers and facilitators influence the implementation and adoption of PrEP in PHSHC?
3. What factors may be related to the successful and equitable implementation of PrEP across PHSHC?

## Materials and methods

### The PrEP-SUO project

This analysis is part of an exploratory implementation science study named “PrEP-suburban Ontario” (PrEP-SUO) using a mixed-methods convergent design. PrEP-SUO was designed to integrate information from qualitative and quantitative studies to identify barriers, facilitators, and implementation strategies, including educational interventions, to increase PrEP adoption at the organizational level and prescribing at the individual level. The PrEP-SUO sample included primary care providers (PCPs) living and working in the northern, southwestern, and central-east regions of Ontario, encompassing remote, sparsely populated districts with significant Indigenous populations, cities with fewer than 100,000 inhabitants, and rural townships. This geographic diversity enabled us to capture perspectives influenced by varying degrees of rurality, population density, and healthcare infrastructure. The research study was approved by the Queen’s University Health Sciences and Affiliated Teaching Hospitals Research Ethics Board (HSREB) (TRAQ# DMED-2650-22).

### Current study

In this manuscript, we present qualitative findings from semi-structured interviews with PCPs and staff working in PHSHC. To identify PCPs working in PHSHC we used purposive sampling to recruit nurses, nurse practitioners, physicians, and sexual health managers, beginning with email invitations sent to all public health units in Southeastern Ontario. To broaden the sample and capture perspectives from clinics serving other geographic regions (e.g., southern and northern Ontario) and underserved populations (e.g., Indigenous communities), we subsequently applied snowball sampling through professional networks across additional Ontario public health units. Participants were sent a letter of information approved by Queen’s HSREB, and provided verbal consent at the beginning of each interview to participate and have the meeting recorded for transcription purposes. Semi-structured interviews were conducted via MS Teams by three researchers between July 2022 and June 2023. Data collection continued until thematic saturation was achieved, with no new themes emerging in successive interviews (26). Each session lasted 30–60 min, was audio-recorded and transcribed using MS Teams, then anonymized and assigned a unique ID. Transcripts were returned to participants for verification; three submitted minor revisions.

### Implementation frameworks

Given its demonstrated relevance to PrEP implementation research (27–32), the PrEP-SUO study was designed using the CFIR as the primary guiding framework to identify facilitators (what worked well), barriers (opportunities to improve implementation), and strategies for scaling up PrEP services (19, 20). Previous studies (27–32) have shown key determinants of PrEP adoption and implementation that mapped very well with CFIR, including intervention characteristics (e.g., simplicity and perceived effectiveness); outer setting factors (e.g., community needs and external partnerships); inner setting factors (e.g., leadership engagement, clinic culture, networking, and availability of resources); individual characteristics (e.g., compatibility with professional roles, knowledge, and skills related to PrEP prescribing); and implementation process factors (e.g., teaming, stakeholder engagement, and collaboration with communities at risk of HIV). All five CFIR domains were integrated into the study design and qualitative analysis. In addition, CFIR domains related to intervention characteristics and individual characteristics—encompassing knowledge, motivation, self-efficacy, and environmental support—were assessed separately through an online survey (33). These findings highlighted limited PrEP-related knowledge and prescribing experience among PCPs in Ontario, as well as key individual-level determinants shaping PrEP provision, including experience serving gender-diverse populations, support from colleagues and clinic teams, and skills and training in sexual health. Together, these results further demonstrate the utility of CFIR for assessing individual-level determinants of PrEP delivery, complementing the organizational adoption processes examined in this study.

To further examine which CFIR domains and subdomains were associated with varying levels of PrEP adoption—and to better understand determinants underlying differences in adoption across clinics—we classified clinics according to their stage of PrEP adoption (knowledge, persuasion, decision, implementation, and confirmation) and adopter categories (innovators, early adopters, early majority, late majority, and laggards), using Rogers’ Diffusion of Innovations framework (DOI) (34). In health services research, this framework is widely used to classify organizations by adoption stage and to distinguish adopter categories reflecting differences in readiness, innovation characteristics, and organizational context. The DOI model has been applied to identify different levels of interest in PrEP in populations (35–37), and at provider levels, demonstrating their relevance for identify key contextual factors related to “rate” of change or uptake (38). Thus, DOI is closely aligned with CFIR and served us to categorize clinics into adoption stages and adoption categories using narratives.

An additional analytic framework was applied to systematically map equity-related factors across PHSHC contexts and CFIR domains and subdomains, using the Health Equity Implementation Framework (HEIF) (25). HEIF is an implementation science framework designed to explicitly integrate health equity considerations into the study of implementation processes and outcomes. HEIF emphasizes how social position, structural inequities, and power dynamics shape the implementation, uptake, and sustainability of health interventions. The framework identifies five equity-salient domains: (1) patient–provider interactions, (2) culturally relevant factors, (3) broader societal and structural context, (4) recipient characteristics, and (5) equity-related implementation processes. By foregrounding these domains, HEIF facilitates systematic examination of how implementation determinants and strategies may differentially affect equity-deserving populations and helps identify pathways to promote equitable adoption and scale-up of interventions across diverse settings. These domains were examined to identify factors likely to influence successful and equitable PrEP implementation.

### Interview guide

We tailored CFIR-based interview guides for each participant group (PHSHC vs. other PCPs), focusing on key constructs influencing PrEP delivery:

1. PCPs’ knowledge, attitudes, and beliefs about PrEP, including effectiveness, complexity, and relative advantage (intervention characteristics);
2. organizational structure, connections with community partners, culture, and implementation climate (inner setting);
3. how organizations address community needs, resources, practice guidelines, and external pressures (outer setting);
4. organizational efforts to adopt PrEP and learn from existing services (implementation process).

The interview guide (Supplementary File #1) was developed by the research team and pilot-tested with two clinical PrEP experts and two providers.

### Data analysis

The analysis presented here was preceded by a hybrid thematic analysis, combining deductive coding informed by CFIR with inductive identification of themes emerging from the data, of interviews conducted with all 28 participants, culminating in the identification of barriers and recommendations for PrEP adoption across all PCPs (26). This initial analysis was followed by a content analysis of interviews with the 18 primary care providers (PCPs) working in PHSHCs, which is presented here (39) and comprised four analytic steps.

First, two researchers developed a summary template for each participant to extract data by interview question, map findings to CFIR domains with supporting quotations, and guide NVivo coding. This summary template was subsequently used to code the interviews in NVivo and was refined into consolidated tables of facilitators, barriers, and opportunities for improvement for each CFIR domain and subdomain across all 18 participants. The second step consisted in the development of an Excel matrix to integrate narratives and summary information for each CFIR domain and subdomain by PHSHC. One researcher with expertise in the CFIR framework reviewed narratives across PHSHCs and applied a color-coding system to indicate the perceived influence of each domain/subdomain of CFIR on PrEP adoption. Green indicated narratives reflecting positive perceptions of PrEP or a favorable influence on its adoption; red indicated negative perceptions or subdomains identified as significant barriers; and yellow indicated neutral or mixed perceptions, or subdomains with minimal influence on implementation. The final matrix with some illustrative quotes is presented in Supplementary File #2. The third step consisted in interpreting the excel matrix by summarizing the influence of each CFIR subdomain based on color distribution: subdomains predominantly coded green were classified as common facilitators (i.e at least six of the 12 of the organizations have a green color); those predominantly red as common barriers (i.e six or more organizations had red color); and those with a mix of green, red, and yellow as contrasting or context-dependent factors. The final analysis consisted in developing an additional Excel-based analytic matrix to identify equity-relevant narratives informed by the HEIF that were not explicitly captured in the initial analysis with CFIR. For this analysis all 18 interviews were revised, recoded and organize using the five equity domains (e.g., patient-provider interactions, cultural factors etc). This analysis was conducted by one of the team members using a similar color-coding scheme as for CFIR to identify barriers, facilitators and contrasting determinants of equity in adoption of PrEP (Supplementary File #3).

### Trustworthiness

Our approach to study design and data analysis integrated constructivist and pragmatic reflexive perspectives. Data were interpreted within the context of current policies, clinical guidelines, and available evidence, while simultaneously prioritizing the generation of actionable findings—specifically, strategies to increase PrEP adoption. Methodological triangulation was employed, including an initial thematic analysis, a team debriefing meeting involving researchers and selected primary care providers (PCPs), and the integration of quantitative survey data to inform and validate recommendations. This process demonstrated convergence between qualitative themes and survey findings, supporting the consistency and credibility of the results presented in this study (22, 26, 33). Interview transcripts were systematically reviewed, and respondent validation was conducted by soliciting feedback from all participants; three participants provided minor clarifications, while the remaining participants offered no additional feedback or did not respond. Dependability was strengthened through independent interpretation of the data by four authors, resulting in consistent identification of key barriers and facilitators. Finally, the interviews generated detailed accounts of participants’ roles and organizational contexts, enabling the development of thick descriptions and supporting the transferability of findings to similar public health settings.

## Results

### Participants characteristics

Of the 28 PCPs participating in the PrEP-SUO study, 18 were working in PHSHC and were eligible for the analysis. These 18 participants worked in 12 PHSHCs linked to nine PHUs across Ontario. Participants included PHSHC managers, public health nurses, and communicable disease or harm reduction managers, with between two and more than 20 years of professional experience (Table S1 in Supplementary File #4).

### PHSHC stages of adoption

Table 1 show the stage of adoption and adoption categories using narratives. Two clinics, identified as innovators, provide comprehensive onsite PrEP services including prescribing and monitoring, often complemented by virtual care partnerships. Three clinics fit within the early adopter category, characterized by strong referral networks and proactive client education without onsite prescribing, as reflected in narratives emphasizing consistent PrEP discussions and partnerships with local clinicians and virtual providers (PHSHC#6, PHSHC#9). Early majority clinics (*n* = 4) rely heavily on external providers with inconsistent referral pathways, providing primarily informational support due to limited capacity, as noted for PHSHC#10. Late majority clinics have minimal engagement with formal referral systems and often assume clients have access elsewhere, with challenges tied to resource limitations and geography articulated through statements about reliance on external specialists (PHSHC#12). Finally, one clinic was identified in the earliest phase of recognition and planning, often due to historically low local HIV risk, as in the case of PHSHC#3.

**Table 1:** Description of the clinics by level of adoption.

Category	Definition	Example descriptions
Innovators, implementation stage	Clinics offering on-site PrEP services (including prescribing and monitoring) combined with flexible service delivery models, such as virtual care partnerships	“In early 2022, we began offering HIV PrEP onsite, providing prescriptions and routine testing. We also collaborate with virtual providers like Go Freddie to tailor to client preferences.” PHSHC#4 Yeah, like I said, exactly like the clinic is part of the sexual health program. PHSHC#1
Early adopters, implementation stage	Clinics with strong referral pathways that are well connected to PrEP providers but do not offer on-site prescribing; varying interest in future on-site implementation. These clinics excelled in building referral networks and linking clients to PrEP providers, often working closely with local clinicians and leveraging virtual services. They also emphasized proactive client education and consistent PrEP conversations.	“We actively ensure clients have PrEP conversations with healthcare providers and connect them to resources. We have a close relationship with local clinicians and virtual providers like Go Freddy, so we have not needed to provide PrEP directly.” –PHSHC#6 “We regularly introduce PrEP in clinic, referring clients to physicians and other clinics better equipped for follow-up.” PHSHC#9 “A recent partnership helped us better identify candidates and consistently discuss and refer for PrEP.” PHSHC#5
Early majority, decision stage	Clinics that refer clients to external providers but lack clear or formal referral systems; informational support is provided, but referral pathways are less consistent.	“Before COVID, we planned to offer PrEP but now refer clients to local pharmacies or online clinics offering barrier-free access.” – PHSHC#2 “We provide informational materials but lack capacity to safely prescribe or follow clients on PrEP.” – PHSHC#10 “There are nearby clinics providing PrEP; duplication of services is avoided at our health unit.” – PHSHC#11 “So we have a prep clinic in [city] run by Doctor [name] and so we do referral for all the individuals who require PrEP” PHSHC#8
Late majority, decision stage	Clinics refer clients but lack established external providers; limited interest in on-site adoption, often due to assumptions about nearby services or low client engagement.	“We provide information about local PrEP clinics but do not offer formal referrals as clinics are usually self-referral.” – PHSHC#7 “Due to lack of local infectious disease specialists, our role is primarily to connect clients with external PrEP resources, which remains challenging.” – PHSHC#12
Laggards, knowledge stage	Clinics with emerging interest in PrEP, currently in early planning stages, with no on-site services or established referral pathways.	“PrEP was not previously on our radar due to low local risk, but recent cases have sparked initial interest and advocacy; we are still at novice to intermediate capacity with PrEP-related work.” – PHSHC#3

Classification based on qualitative interviews.

### PHSHC organizational characteristics

Tables S2–S5 in Supplementary File #4, show additional description of the clinics. Geographically, the clinics span a wide area across Ontario, encompassing mostly rural regions (e.g., PHSHC#3, PHSHC#12) as well as mixed urban–rural settings (e.g., PHSHC#2, PHSHC#9). Clinic sizes vary considerably, from a single nurse practitioner (PHSHC#3) to larger teams of 10–11 nurses (PHSHC#5). Service delivery modalities also vary: many clinics offer a combination of online, community outreach, and in-person services, with some integrating telephone and chat platforms (e.g., PHSHC#1, PHSHC #4, PHSHC #9 and PHSHC 2). Core sexual health services such as STTBI management and HIV prevention/testing are widely available, while additional offerings include birth control, harm reduction programs, pregnancy counseling, and tuberculosis services in select locations.

Organizationally, most clinics align their vision and mission with HIV prevention and public health goals, with some placing an explicit emphasis on equity and addressing social determinants of health (PHSHC#8, PHSHC#10). The majority actively collaborate with local non-governmental organizations, harm reduction programs, and laboratory services, with some extending partnerships regionally or through academic institutions. A few clinics, however, lack these wider connections, potentially limiting access to additional resources (eg, PHSHC#2, PHSHC#7, PHSHC#10).

Leadership engagement and access to up-to-date PrEP knowledge are common in clinics offering on-site services (Supplementary Table S2). Conversely, several clinics face gaps in resources, laboratory support, or formal medical directives needed to scale PrEP provision. Notably, rural and smaller clinics (such as PHSHC#3 and PHSHC#12) encounter greater challenges related to staffing, infrastructure, and resource availability. Staff generally demonstrate enthusiasm for adopting new methods and report allocated time for training, though most clinics acknowledge ongoing needs for PrEP-specific education, with the exception of PHSHC#1 and PHSHC#4, where training needs are less pronounced.

### Main determinants of adoption and equitability

Using the CFIR and the HEIF, we identified three common facilitators, five common barriers and four contrasting factors across PHSHC, all of which have important effects on equitable adoption of PrEP. Tables 2–4 summarize the interpretation according to domains and subdomains of CFIR, while Table 5 through domains and subdomains of HEIF.

**Table 2:** Summary of common facilitators to PrEP adoption by CFIR domain and subdomain.

Common facilitators	CFIR domain/Subdomain	Summary of the interrelation
Strong support and belief in PrEP as an effective HIV prevention tool	Intervention characteristics (Intervention source and effectiveness)	Participants have a positive perception of PrEP, its effectiveness and safety, most of this driven by the knowledge and awareness of guidelines and directives in their organizations
	Outer settings (Guidelines and policies)	
Partnerships and networking:	Outer setting (Partnerships)	The integration of PrEP services within PHSHCs was heavily reliant on effective networking. Diverse referral pathways, strategic partnerships, create a flexible and adaptive framework that accommodates client needs while mitigating operational burdens. Moreover, strong advocacy within these networks provides the necessary momentum to overcome challenges
	Inner setting (Networks)	
	Intervention characteristics (Adaptability, flexibility)	
	Process Engaging	
Organizational structure, services, and culture	Inner setting (Structure, climate, culture)	The structure, organizational climate, and compatibility of PrEP with mission and vision of the organization are key facilitators, the public health standards support this understanding of their role in HIV prevention
	Outer setting (Policy and laws)	

**Table 3:** Summary of common barriers to PrEP adoption by CFIR domain and subdomain.

Common barriers	Domain	Summary of the interrelation
Recovery from COVID-19	Outer setting (Critical incidents)	COVID-19 pandemic significantly disrupted PrEP services through staff redeployment and service delays, affecting the human resources available for expansion of services. Pre-existing policies and adaptive strategies have aided recovery, though challenges in rebuilding capacity and staffing persisted.
	Inner setting (Available resources)	
Limited Access to Primary Care and Reliance on Alternative Services	Outer setting (Local conditions)	Local PCPs often show little interest and limited capacity to offer PrEP, making it difficult for PHSHCs to connect clients with needed services. To address these barriers, some organizations are raising community awareness and adopting online care models to bridge the geographical gap and expand PrEP access.
	Inner setting (Available resources)	
	Process (Engagement)	
Insufficient funding for PrEP coverage	Outer setting (Funding)	Lack of policy for a free PrEP access limit feasibility and accessibility of populations, and increase perception of the complexity of the program, as staff noted the need to help navigate clients for funding.
	Inner setting (Complexity)	
Organizational constraints	Inner setting (Available resources)	Lack of human resources within the clinics limit interest in provision of PrEP in a more innovative ways, this relates to unknown operational cost and the need of having assessments to identify if the service is needed and its cost.
	Intervention characteristics (Operational cost)	
	Process (Need assessment)	

**Table 4:** Summary of contrasting factors related to PrEP adoption by CFIR domain and subdomain.

Contrasting factors	Domain	Summary of the interrelation
Demand for PrEP	Intervention characteristics (Relative advantage)	Participants recognize PrEP as a vital tool against HIV, but they differ on how urgently to expand its services. Variation in perceived urgency influenced readiness for implementation. A few called for more community needs assessments to guide resource allocation
	Inner setting (Tension for change)	
	Process (Needs assessment)	
Workflow integration	Intervention characteristics(Complexity/Characteristics)	Integration of PrEP into routine clinic workflows depended on balancing operational complexity, staffing capacity, and follow-up requirements. While some clinics viewed these demands as prohibitive, others leveraged flexible models—such as referral pathways—to manage complexity. Leadership support and alignment with clinic workflows facilitated integration. External partnerships were used to offset internal capacity limitations.
	Inner setting (Leadership, compatibility)	
	Outer setting (Partnerships)	
Local conditions	Outer settings (Local context, Financing)	The social barriers of equity deserving populations in some areas limited the interest of staff in the clinics. Addressing the social determinants of health is a more urgent need.
	Inner setting (Relative priority)	
Learning climate and current knowledge	Inner setting (Learning climate and culture)	There is a commitment to learn and increase capacity to offer PrEP, however, some expressed the need of further assistance to do it.

**Table 5:** Main determinants of equity adoption of PrEP across PHSHC using HEIF.

Determinant	Sub-determinant/Theme	Core findings
Patient–provider interaction	Strong support and belief in PrEP as an effective HIV prevention tool (facilitator)	Strong provider endorsement helped normalize PrEP and supported engagement among clients who experience stigma or mistrust of health systems
	Creating safe, client-centered, and inclusive clinic environments amid persistent systemic barriers (facilitator)	Trust, confidentiality, and stigma-free care positioned PHSHCs as key access points for equity-deserving populations, particularly those concerned about disclosure or discrimination.
	Limited interest and knowledge among PCPs outside PHSHCs (barrier)	Limited external prescribing capacity may disproportionately affect rural, transient, and structurally marginalized clients who rely on public health clinics for HIV prevention.
Culturally relevant factors	Persistent stigma and misconceptions impacting PrEP uptake (barrier)	Stigma and misinformation may disproportionately limit PrEP uptake among people who experience higher levels of stigma, such as PWUD, migrants, Indigenous communities, and individuals experiencing homelessness.
	Limited awareness and knowledge gaps hindering PrEP uptake and delivery (barrier)	Knowledge gaps (at individual, community and provider levels) contributed to inequitable access by reducing demand and delaying engagement among populations with limited access to information channels.
Societal and structural context	Limited geographical access to primary care and reliance on alternative services (barrier)	Provider shortages exacerbated inequities, even among those relying on public health services. When populations do not access PHSHC, their awareness and access to PrEP tend to be lower, particularly in communities with limited access to information channels.”
	Financial barriers and limited coverage impede PrEP access and adherence (barrier)	Cost barriers affect all without insurance, and lack of insurance is disproportionately present in low-income and precariously insured populations, reinforcing inequities in PrEP access and retention.
	Geographical and service delivery barriers in a large, dispersed region (barrier)	Rurality and geographic dispersion exacerbated inequities, contributing to delayed initiation, fragmented care, and reliance on referrals to urban centers.
Recipient characteristics	Diverse client needs and social challenges (barrier)	PrEP uptake was influenced by individual risk perception, health literacy, stigma, and social instability.
Equity-related implementation processes	Limited local PrEP services and challenges in targeted health promotion (barrier)	PrEP services and eligibility frameworks remained largely centered on GBM, despite rising HIV risk among PWUD and people experiencing homelessness. Capacity for targeted health promotion was limited. Narrow service focuses risked perpetuating inequities by underserving populations with growing HIV vulnerability.
	Collaborative, outreach-focused approaches enhancing PrEP access and support (facilitator)	Outreach-based, partnership-driven models were critical for reaching underserved populations, though variability in funding and collaboration limited consistent equity-oriented implementation.
	Holistic, client-centered care and tailored support for priority populations (facilitator)	Holistic and tailored approaches supported more equitable access, particularly for populations facing intersecting social and structural barriers.

#### Common facilitators

##### Strong support and belief in PrEP as an effective HIV prevention tool

Participants across PHSHCs were all aware of PrEP as strategy for HIV prevention, and consistently expressed strong, positive beliefs in its effectiveness. PrEP was widely recognized as an evidence-based intervention and, in some clinics, routinely integrated into client counseling practices. These views were reinforced by ongoing updates from regional studies, Canadian guidelines, and Public Health Ontario, which strengthened confidence in the evidence supporting PrEP.

*It’s actually quite highly effective [referring to PrEP], and we’ve definitely seen evidence of that locally with [neighboring Public Health Unit] monitoring their HIV rates after starting (PHSHC#10)*.

*I think I do have general awareness, as well as my team… especially increased over the past couple weeks with the real push behind the new HIV guidelines (PHSHC#7)*.

There was a common understanding among participants that PrEP deserves greater visibility as a vital public health strategy, and this shared conviction served as a powerful facilitator for equitable implementation. Strong provider endorsement of PrEP as effective prevention intervention helped normalize PrEP and supported engagement among clients who experience stigma or mistrust of health systems.

*I think everyone who knows that they're going to be involved in risky behaviors Whether you're female, male, or whatever you identify as will benefit from Prep, because again, it's a prevention strategy regardless of what you want to do in your life (PHSHC#11)*.

##### Partnerships and networking

Networking plays a crucial role in enhancing the provision and flexibility of PrEP services across many PHSHCs, especially those relying on external providers. This flexibility allows services to be tailored to client preferences, whether through pharmacies, referrals to their primary care doctors, other PrEP providers in the community or virtual care providers (Table 1). Robust networks also extend beyond healthcare providers to include community-based organizations, other public health units, harm reduction programs, and regional bodies. Although the strength and formality of these collaborations vary—with some being informal—they collectively lay the foundation for more structured service delivery. For example, a participant detailed proactive outreach to secure expert guidance, formal education efforts, and connections with qualified prescribers:

*I reached out to HIV and Hepatitis C programs which connected me with OHTN [Ontario HIV Treatment Network] and prescribers, including an ID [Infectious Disease] specialist and a nurse practitioner, while OHTN supports formalizing our education (PHSHC#3)*.

Importantly, many of these partnerships enable clinics to reach underserved populations, address their unique needs, and link them to both social and health services. There is a notable interest in shifting from fixed clinic-based models toward outreach and mobile services targeting harder-to-reach groups such as PWID and Indigenous communities. One participant narrative corroborated the efforts of clinics for linkage with key community partners for reaching those in need:

*So we have, for example, clinics that provide testing to increase uptake of testing amongst men who have sex with men and under housed individuals. we have a partnership with our local aid service organization that we provide clinics at as well as some of our female orientated shelters in the community (PHSHC#8)*.

##### Organizational structure, services, and culture

Participants consistently emphasized organizational cultures grounded in respect, comprehensive care, and a strong commitment to serving priority populations. These values were reflected in deliberate efforts to address social determinants of health and to build trust with communities, factors participants identified as central to effectively reaching underserved groups.

*We definitely know there is increased risk in the community. While we may not be able to solve all issues like housing or addiction, connecting people with PrEP is a concrete way to reduce their risk. Public health plays an essential role in providing these services (PHSHC#3)*.

Participants also described positive client feedback, which they attributed to organizational values that prioritize diversity, inclusion, and non-judgmental care. Clinics actively strive to create LGBTQ+ affirming environments, fostering safe spaces for testing, counseling, and prevention discussions.

*We had some good feedback that we were a very LGBTQ+ friendly organization (PHSHC#2)*.

These welcoming settings were seen as critical for engaging clients who may otherwise avoid care due to prior experiences of stigma or discrimination. Providers further emphasized a commitment to individualized, client-centered care that respects autonomy and avoids stigmatizing labels. This approach reflected a broader shift away from narrow or stereotypical constructions of HIV risk and toward empowering clients to make informed decisions about PrEP. Trust, confidentiality, and stigma-free care thus positioned PHSHCs as key access points for equity-deserving populations, particularly for individuals concerned about disclosure. One participant highlighted this intentional effort to challenge stigma:

*You know, initially there were some stereotypes about who needs PrEP. We work very hard in our clinic to keep open minds and put aside those barriers of stigma about who needs PrEP (PHSHC#9)*.

Finally, participants underscored the importance of holistic and tailored approaches to care, especially for clients facing intersecting social and structural barriers. Providers described taking time to understand each client’s broader context and priorities as essential to supporting equitable access and sustained engagement.

*There are so many layers, especially related to social determinants of health, that create barriers to access. We strive to take a holistic approach—understanding where each client is at and what their current needs are—to support them in achieving optimal health (PHSHC#5)*.

#### Common barriers

##### Recovery from COVID-19

Most clinics are recovering from COVID-19 disruptions. Participants reported that COVID-19 created major barriers by disrupting service delivery and delaying PrEP availability.

*We actually had our PrEP policy and procedure ready to go in 2019 and then the pandemic hit (…) So we didn't begin offering it until 2022 (PHSHC#4)*.

Participants described pandemic adaptations such as phone follow-ups with nurses and doctors, and new referral and testing pathways through local labs. Pre-existing PrEP policies helped services resume more quickly after emergency measures were lifted. Only one PHSHC prioritized the delivery of PrEP services over other sexual health clinic services during the pandemic. Most narratives indicated clinics were in recovery, adjusting services, revising schedules, and reopening programs:

*We didn’t have the capacity to be running all of the clinics in the area. We were dedicated to keeping our clinics open, but we did have to change our delivery model (PHSHC#10)*.

Staff redeployment and emergency response demands placed significant strain on HIV prevention efforts, resulting in clinic closures, reduced operating hours, and decreased flexibility in service delivery. Participants reported that these disruptions interrupted routine HIV testing and timely access to treatment, which they perceived as contributing to an increase in local HIV diagnoses.

*Well, the most recent change in our clinic is in many ways, they were very limited and we're restricted during COVID and which I think It was a mistake we have seen a rise in HIV cases in our area. There was lack of access to treatment testing. We have people who do regular testing that was no longer available (PHSHC#2)*.

##### Limited access to primary care and reliance on alternative services

Participants noted that local PCPs outside the organization showed little interest or capacity to offer PrEP, posing considerable challenges for PHSHCs relying on external referrals:

*A client wants to access treatment with their family healthcare provider. We'll be trying to pressure their family healthcare provider to prescribe them PrEP. And I have heard that they've had challenges accessing PrEP (PHSHC#6)*.

This scarcity of willing providers hinders the effective distribution of PrEP, exacerbating barriers for underserved populations, particularly those in rural areas. These populations face additional barriers like long travel distances and inflexible appointment systems that limit access to sexual health services:

*Most of our family doctors work at a clinic outside of town, but you book by appointment. You have to drive to get there. So they're not seeing the population that needs to be seen (PHSHC#3)*.

In response to these challenges, some organizations have adopted creative strategies, including efforts to raise community awareness of PrEP, while other clinics suggested the adoption online or hybrid care models to bridge geographic gaps and improve access, especially for those without a primary provider:

*We have a high proportion of folks who don't have a primary healthcare provider. I think having online care such as that or just for other things too… it's hugely beneficial in a rural, remote type area (PHSHC#7)*.

##### Insufficient funding for PrEP coverage

Persistent financial barriers undermined perceptions of PrEP’s feasibility and access for most participants and impacts adherence. One participant draws a parallel to the high costs some people living with HIV face for antiretroviral medications, noting that this financial burden leads some individuals to discontinue their medication, resulting in poorer health outcomes and increased risk of transmission and secondary infections.

*We've had people [living with HIV] stop their medication because they couldn't afford it even after being on Trillium. It would still cost $400.00 or $500.00 a month, so they stopped taking it and then, of course, they're infectious and other infections can stem from there. They're sick. They're in the hospital (PHSHC#2)*.

Cost barriers affect all without insurance, and lack of insurance is disproportionately present in low-income and precariously insured populations, reinforcing inequities in PrEP access and retention.

*I would say negative would be just the ability for everybody that needs it to access it because I think right now you know it is accessible if you have a health plan accessible if you have one but there's definitely those that are not on that right now that don't have that access (PHSHC#5)*.

Participants noted that limited insurance coverage increases the need to navigate financial assistance options for PrEP clients, especially among Indigenous and other equity-deserving groups (e.g., immigrants, low-income). This gap often requires organizations to help clients secure ongoing support, which can feel like unclear without pre-determined processes.

*So, we would want to be aware of that and cognizant of, umm, you know, the financial uh aspect in regards to our clients and then what that would potentially mean for us? Is that something that we would have to look at helping cover or things like that, right? Because it's great to have this all done and put in place. And then it's another thing where if people can't actually afford it (PHSHC#10)*.

Participants recognized the importance of a robust national policy to facilitate PrEP accessibility, drawing parallels with the successful approval and coverage of treatments for other conditions like Hepatitis C.

*People can be covered for their Hep C treatment. So I'm hoping that we can do that with PrEP (PHSHC#12)*.

##### Organizational constraints

Participants identified major organizational barriers to PrEP implementation, including the need for more prescribers, nursing staff, lab support, and operational funding:

*We don’t have any dedicated sexual health physicians that are working with us. So, we have our medical officer of health, and (…) the odd [medical] resident (…). But we don’t have any hours of service to facilitate the ongoing thing that would need physician support. (PHSHC#2)*.

They emphasized that understanding PrEP implementation costs is key to assessing feasibility and guiding decision-makers. Accurate estimates and new funding were seen as essential for program growth.

*I think that's a hard one to answer (re: cost). I mean there would be administrative costs, there would be staff costs (…) I'd say it would probably take one FTE of a public health nurse (PHSHC#2)*.

The heavy reliance on stagnant provincial and municipal funding also makes it difficult to secure resources for additional staff:

*Without those additional provincial funded dollars, that is then coming out of a municipal levy (…). Where are those financial human resources support? Is there separate funding for this? (PHSHC#8)*.

##### Individual characteristics: providers and client’s social barriers

Participants acknowledged that PrEP uptake and clients served by clinics remain concentrated among GBM, with limiting reach among uninsured populations, PWUD (among whom some noted rising HIV risk) and individuals experiencing homelessness.

*And those clinics were very well attended. Uh, we had great feedback from clients. People loved those clinics. But what we noticed when looking at the data was a fair number of them did have family doctors, a fair number of them were students who had access to student services (PHSHC#1)*.

Misconceptions and PrEP related stigma remain common among both clients and decision-makers, including beliefs that PrEP is only for people living with HIV. Concerns about side effects, cost, and social consequences further deter uptake, particularly among marginalized populations who may distrust healthcare interventions.

*Umm, I do know that there is a huge stigma to it and that's why a lot of a lot of clients are either unwilling to receive PrEP or simply are unaware of what prep is or where it's available. Or when they can actually have PrEP. I think a lot of misconceptions are you can only receive treatment if you have HIV (PHSHC#11)*.

Among providers, participants noted knowledge gaps (at individual, community and provider levels) contributed to inequitable access by reducing demand and delaying engagement among populations with limited access to information channels.

*You know, I don't think it's that well known amongst many decision makers that such a thing even exists, you know. Just generally like that's my take on it (PHSHC#1)*.

#### Contrasting factors

##### Demand for PrEP

Participants stressed PrEP’s crucial role in fighting HIV, though urgency to expand services varied. One participant strongly advocated for on-site PrEP rather than continue to the referral delivery,as they notice importance to boost uptake among GBM and vulnerable groups like sex trade workers.

*I would like to be able to offer PrEP at the public health unit. That's for a number of reasons. Before COVID, we were seeing quite a lot of GBMSM people at risk of HIV. We have some sex trade workers as well… Running the service on the spot is probably the best way when someone’s interested, to be able to provide that service for them right away as opposed to saying, ‘Oh, just go over there, here’s that number’ (PHSHC#2)*.

Other participants anticipated growing PrEP demand due to rising syphilis rates and sexual health challenges. One participant noted that although community awareness is low, increasing of sexually transmitted infection rates indicate demand will rise:

*I think PrEP, in general, is not very known to the community, but given the number of syphilis rates and, you know, the sexual health rates in Ontario, it’s only a matter of time (PHSHC#11)*.

Conversely, some participants questioned the urgency of expanding PrEP. One participant cited existing referral pathways and low local HIV rates as reasons widespread implementation may not be immediately needed:

*Given the epidemiology that we're seeing*—*with really high syphilis rates and the changes in multidrug resistance*—*we have very low rates of HIV in (city) (PHSHC#8)*.

Echoing above comment, another participant urged caution, calling for more community needs assessments before expanding PrEP:

*We don’t see a lot of need for it right now, so in order to actually think that would be a valuable piece to implement, I think we need to first go and do surveys in the community or something like that to really identify… Is this a gap that should be filled by public health? (PHSHC#7)*.

A needs-based approach was evident in an “innovator” clinic. One participant described how customer satisfaction surveys and engagement with community HIV partners informed the decision to offer PrEP:

*We started to see that in some of our customer satisfaction surveys, that PrEP was something we should be looking at. That, coupled with consulting with our community partners in HIV as well, led us to decide to start looking at PrEP (PHSHC#4)*.

##### Workflow integration

Differences emerged in how clinics perceived the integration of PrEP into their current workflows. For some clinics, adding PrEP meant reconfiguring workflows, making feasibility challenging. One participant noted that integration requires accessible medical directives, qualified prescribers, and staff time to offer PrEP on-site.

*For us, it really does continue to be resources. So it would be establishing those partnerships and both funding and human resources to be able to do that (PHSHC#8)*.

In contrast, clinics using referral systems found integration more manageable, with some likening it to prescribing birth control:

*So we're treading gently, but positively to help people understand that it's quite an easy process* (…) *if you prescribe the birth control pill, you can prescribe… you know, I keep coming down to that because we really want it to be an easy thing. A directive (PHSHC#9)*.

Innovator clinics noted that adding PrEP increased workload with new tasks like managing follow-ups, labs, and social determinants of health; obstacle that in both cases was managed with the hiring of a PrEP coordinator:

*It does add work to our clinic coordinator (…). Each of our clinics has a clinic coordinator, who is a public health nurse who also sees clients in the clinic. They have the additional role of managing lab reports, following up by calling clients, booking them in if they have positive STI results (PHSHC#4)*.

##### Local conditions

Across clinics, social and structural conditions were consistently identified as limiting both access to and sustained engagement with PrEP. However, participants from one clinic described challenges in providing consistent PrEP care to individuals experiencing homelessness, for whom unstable housing and limited access to healthcare services disrupt medication adherence and routine follow-up. As one participant explained:

*A lot of the increases that we're seeing locally in terms of our case numbers are related to people who use injection drugs and are currently underhoused—the homeless. So in terms of the inability to maintain daily medications and access to a daily medication, it tends to be challenging here (PHSHC#3)*.

A second participant emphasized how racism and discrimination within the local context generate fear and mistrust of healthcare systems, further discouraging individuals from seeking services—barriers that would likely extend to PrEP uptake and continuity of care:

“*There’s a lot of stigma and discrimination in [city]. It’s bad here. The racism is really pretty horrific here” (PHSHC#3)*.

Rurality and geographic dispersion exacerbated inequities, contributing to delayed initiation, fragmented care, and reliance on referrals to urban centers.

*We also know that because of where we are geographically, people do have barriers. To accessing PrEP and we know also that people's primary care providers from what we hear anecdotally from our clients, a lot of people are not comfortable even requesting this from their family doctors or their family. Doctors are simply not comfortable prescribing it or getting involved (PHSHC#10)*.

##### Learning climate and current knowledge

The need for additional training and the organizational climate for learning varied across clinics. For some participants training is an ongoing process and they do it to better meet client needs:

*Education is an ongoing process here and we're keen to learn as much as we can, so we know that PrEP is certainly effective and available and easy to get and affordable in many situations and our next focus is really to be getting awareness out into the community a little bit more (PHSHC#9)*.

*We're all very motivated to promote PrEP, and you know, we have it on our documentation tool—it's part of our practice. If we're having a client with certain infections, we're making sure to discuss PrEP (PHSHC#5)*.

Some had no direct PrEP training but noted opportunities within organizations that support continuous learning:

*We have not had anything specific to HIV care or PrEP in terms of training, the resources that were oriented to when we're hired basically as they're updated by whoever's providing them, then you're expected to review the new documents and attend webinars and education as they pop up (PHSHC#3)*.

Other participants refer that their clinics does not have specific time devoted to training and most of the training came from external initiatives such as those to get updated in guidelines.

*So at this point it's more of the prevention, what's your best treatment and what are the guidelines. And like I said, we're always up to date with that would depending on what the ministry sends us and informs us about (PHSHC#11)*.

## Discussion

This study is the first in Canada to comprehensively examine factors influencing PrEP adoption in public health–led sexual health clinics (PHSHCs), providing novel insight into determinants of both adoption and equitable PrEP delivery. Participants represented 12 PHSHCs across 9 of Ontario’s 34 public health units at the time of the study, with a focus on small and mid-sized health unit regions, and encompassed a broad range of roles, including direct patient care, mid-level management, and senior leadership. Participating clinics varied substantially in their social, geographic, and resource contexts, as well as in their stages of PrEP adoption. Five clinics were classified as active implementers (Innovators or Early Adopters), either offering onsite PrEP services or maintaining strong, formalized referral pathways. One clinic was positioned at an early stage of the innovation–decision process, characterized by emerging awareness and interest in PrEP. The remaining seven clinics relied primarily on informal referral practices and had not yet chosen, or had not expressed commitment, to offer PrEP onsite. This heterogeneity enabled the identification of both common facilitators and barriers across clinics and, importantly, highlighted individual-, contextual-, and organizational-level factors associated with distinct adoption categories, as discussed below.

### Facilitators, barriers and contrasting factors

Consistent with CFIR, several *facilitators* of PrEP adoption emerged from the interview data. Participants demonstrated strong knowledge of PrEP, positive beliefs regarding its effectiveness in reducing new HIV infections, and a clear interest in further PrEP-related training, reflecting favorable intervention characteristics. Additional facilitators included clinics’ strong sexual health mandates and staff expertise, cultures emphasizing respect and comprehensive care (inner setting), deep engagement with and understanding of local communities (outer setting), and well-established partnerships (inner and outer settings). These facilitators have been documented previously in adoption of PrEP in sexual health clinics in Ontario (10, 18) and in other settings (19, 29, 31).

At the same time, persistent multilevel *barriers (outer and inner setting)* constrained PrEP implementation. A commonly cited barrier across clinics was the COVID-19 pandemic, which resulted in widespread resource redeployment. At the time of interviews, many public health units were still in recovery mode, reorganizing staffing and reopening suspended services (40). The pandemic also accelerated PCPs’ retirements, further constraining clinical capacity. Relatedly, many PHUs identified limited resources (inner setting)—including nurse time, prescribers, and dedicated funding—as key reasons for not offering on-site PrEP services. Beyond pandemic-related disruptions, our earlier analyses also identified structural funding constraints and limited leadership prioritization of PrEP (inner settings) as additional barriers to implementation (26). Participants suggested several strategies to address these challenges, including task shifting, targeted funding initiatives, and clearer guidance on PrEP logistics and costs (26). Improved understanding of PrEP delivery requirements may help alleviate concerns, as on-site PrEP services may be less resource-intensive than initially perceived. Notably, clinics that had successfully implemented on-site PrEP services often relied on dedicated PrEP coordinators, highlighting the importance of additional human resources to support sustainable implementation.

Another important barrier was the limited availability and willingness of PCPs (mainly those external to the organizations) to oversee PrEP prescribing (19, 41). Structural factors, including funding constraints, limited provider motivation, persistent stigma, and low awareness or knowledge of PrEP, have contributed to the small and geographically concentrated pool of PrEP prescribers in the province (26, 33, 41). In Canada, PrEP prescribing requires a licensed prescriber, and while some PHSHCs have established external partnerships with prescribers (e.g., virtual providers, in-person clinicians, or select pharmacies), these arrangements were not consistently successful across all clinics. Expanding on-site prescribing capacity—particularly through nurse practitioners—was viewed by the sample of PCPs in our study as a promising approach to enable more direct and timely PrEP delivery (26), as demonstrated by clinics in Toronto and Ottawa (18, 42).

Another key finding relates to *contrasting determinants*—that is, factors that differentiated clinics by their stage of PrEP adoption. These included perceptions of local PrEP demand (inner setting/process), the ability to integrate PrEP into existing workflows (inner setting), and the availability of supportive learning environments (inner setting). Perceived demand varied across settings: for some clinics, rising syphilis rates reinforced the need for on-site PrEP services, whereas others deprioritized PrEP due to low local HIV incidence. While some clinics emphasized the importance of conducting formal needs assessments, clinics characterized as innovators had already completed these assessments and acted on their findings. Perceptions of need were shaped by local epidemiology, social factors, and prevailing clinical guidance. Views on workflow integration also diverged. Clinics in advanced stages of PrEP adoption reported that existing service structures could accommodate PrEP delivery, whereas others anticipated substantial challenges related to follow-up, laboratory monitoring, and addressing clients’ social needs. These differences likely reflect variation in infrastructure, staffing, and resource availability across PHSHCs.

Finally, clinics differed in their confidence and comfort in providing PrEP, which appeared closely linked to disparities in training opportunities and learning environments. Clinics with more advanced PrEP implementation described routine incorporation of PrEP-related education and skill-building into their activities, whereas others reported limited access to training. Training needs and related recommendations have been detailed in our previous work (33). Expanding training for clinic staff and external providers is critical, given gaps in knowledge, skills, and motivation among PCPs (26, 33). Training should be adapted to provider characteristics and perceptions (43).

### Equity-related determinants of PrEP adoption

There are important findings related to determinants of equitable adoption of PrEP services. Participants acknowledged that while PHSHCs effectively reach a key priority population—GBM—they are less consistent in reaching other populations, including PWUD, Indigenous communities, and individuals experiencing homelessness. In Ontario, approximately 90% of PrEP users are GBM, and recent studies have documented substantially lower uptake among Indigenous peoples, immigrants, and other racialized communities (44–47). The current research, as well as others (2, 48, 49), noted that these populations experience higher levels of stigma, poverty, and structural barriers, including difficulties with daily medication adherence, attending appointments and laboratory testing, and accessing prescriptions. Among PWUD, challenges such as homelessness and co-occurring mental health conditions were highlighted by our participants as further diminishing their interest in expanding PrEP delivery. Despite these barriers, PHSHCs reported actively seeking to address inequities through tailored strategies and partnerships with community organizations to improve access to PrEP for underserved populations.

Medication cost also emerged as a persistent and significant barrier to equitable PrEP adoptio (11, 41, 50), operating across multiple levels of the healthcare system. Providers consistently reported that clients delayed initiation, interrupted use, or discontinued PrEP altogether due to out-of-pocket costs and ongoing financial strain. At the provider level, participants described hesitancy in routinely recommending PrEP, particularly for patients with unstable employment or precarious immigration status, given uncertainty around coverage eligibility and reimbursement pathways. Organizationally, several participants perceived PrEP delivery as logistically and financially unfeasible for individuals without private insurance, limiting proactive outreach to populations most affected by HIV. These multilevel cost-related barriers contributed to selective offering of PrEP and reinforced reliance on fragmented funding mechanisms, placing an additional burden of navigation on both clients and providers. As a result, participants from PHSHC recognized that the current PrEP funding model in Ontario risks reproducing—and potentially exacerbating—existing inequities in access, particularly among racialized communities, newcomers, young people, and individuals with unstable income or housing (14, 15, 17, 28, 51). Participants emphasized that without clearer, more consistent public coverage and streamlined navigation supports, cost will continue to undermine equitable PrEP uptake, engagement and sustainability.

Rurality emerged as a key contextual factor that further constrained the capacity of PHSHCs to offer PrEP services in, or serving, predominantly rural areas. In these settings, participants described multiple intersecting factors that deter PrEP adoption. Rural clinics were reported to serve populations facing compounded vulnerabilities, including higher levels of racialization and poverty, which exacerbate existing barriers to PrEP access. Consistent with prior studies, participants also noted limited provider availability, as well as gaps in provider knowledge and interest in PrEP (52). High levels of stigma, racism, and other forms of discrimination were likewise reported, echoing findings from other contexts (53, 54). Overall, these findings align with previous literature and our earlier thematic analyses (26), which document persistent inequities between rural and urban areas in Ontario and beyond.

### Learning points and recommendations

Although referral arrangements were perceived as highly functional, many were informal and inconsistently structured. Importantly, PHSHCs do not systematically track referral outcomes, leaving the effectiveness of these pathways largely unknown. Notably, many clinics, including early adopters, expressed limited interest in on-site PrEP provision, citing strong external partnerships, staffing constraints, and workflow limitations. However, referral-based models have shown mixed outcomes in other contexts (55, 56): while they may streamline access, they can also introduce barriers to completing referrals and obtaining prescriptions. In contrast, integrating PrEP into sexual health clinics offers several advantages, including timely access to specialized care, additional diagnostics, and improved program sustainability, potentially reducing client attrition. Based on these findings, we recommend that early adopter and early majority clinics consider a temporary “one-stop shop” or same-day PrEP model during initial engagement, with transition to external providers for ongoing care. Additionally, incorporating case managers or coordinators, as well as PrEP navigators, and ensuring financial coverage may improve referral outcomes (57).

PrEP implementers in PHSHCs shared strategies that supported adoption, including partnerships with external providers, local needs assessments, implementation planning, and capacity building through training and medical directives, echoing findings from other studies (32, 58–60). Clinics that successfully integrated PrEP showed strong adaptability to organizational challenges, particularly service reductions and staff redeployments during COVID-19, as seen elsewhere (61). One clinic, for example, shifted to telehealth, online lab requisitions, and referrals to online providers (22). This adaptability to external conditions and population needs is a known determinant of successful PrEP adoption (62). Participants also stressed the need to understand PrEP-associated costs to assess feasibility and guide decision-making. PHSHCs with existing programs can help reduce barriers by sharing workflows, training materials, and guidance. To support broader on-site PrEP delivery, our previous analysis with all PCPs in the PrEP-SUO and prior research highlighted several key strategies: incorporating a PrEP prescriber, adapting medical directives or standard operating procedures, conducting needs assessments, enhancing nursing staff training, and undertaking comprehensive financial planning (22, 26, 60, 61).

Equity related considerations are further needed in planning the adoption and scaling up of PrEP in Ontario. Participants noted that their services often fail to reach and meet the needs of certain groups (underhoused, Indigenous people, PWUD). These gaps were further compounded by competing service priorities between addressing social versus sexual health needs, as well as by local contexts of stigma and discrimination. Strengthening organizational readiness—through the identification of PrEP champions, investments in staff capacity building, meaningful community engagement, and training of local primary care providers—will be essential to advancing equity in PrEP delivery (63, 64). Complementary strategies proposed in the literature include the provision of free PrEP, integration of harm reduction services (the one-stop shop model), and interventions that address broader social conditions, such as housing instability (60, 65–68). Reaching unhoused populations for HIV and STTBI testing as has been done by some PHSHC, may offer scalable solutions for offering same day PrEP (47). Pharmacy-based PrEP delivery models would offer another opportunity to increase access to prescribers, with potential implications for access, adherence, and service integration, especially in rural areas (69–72). Implementation of injectables could offer a potential for addressing adherence, and it is considered as an option for PWUD in Canada (73); although their impact in decreasing access and uptake of PrEP is questioned (22, 74, 75). Lastly, telehealth delivery of PrEP, expanding the role of nurse practitioners and adopting task-shifting approaches have shown to be effective, acceptable and sustainable in other Ontario clinics (65, 68).

### Strengths and limitations

This mixed-methods study captures perspectives from a diverse group of providers across 12 PHSHCs in Ontario, reflecting a wide range of experiences and organizational contexts. Notably, it includes input from sexual health managers responsible for program planning and resource allocation—a group often underrepresented in PrEP implementation research, which typically focuses on providers and patients. However, our sample primarily includes providers with a strong interest in and favorable views of PrEP, which may limit the generalizability of findings to providers outside public health or PHSHC settings, who may be less inclined to adopt PrEP. In addition to the content analysis conducted here, we conducted a thematic analysis of all the interviews, identifying themes aligned with the CFIR framework, thereby enhancing the trustworthiness of our findings. Methodological rigor was further supported by strategies such as researcher expertise, thematic corroboration, member checking, independent analysis, and rich contextual descriptions to ensure credibility, dependability, and transferability.

The use of the CFIR represents a key strength of this study, as it provides a comprehensive and systematic lens to examine contextual determinants of PrEP adoption across 12 clinics and to generate actionable recommendations informed by sites with successful implementation. Although our analytic approach relied on a simplified coding scheme using three categories—which may appear limited given recent recommendations for more nuanced CFIR scoring systems—we found that this approach was appropriate for the scope and sample size of the study. In the context of 12 clinics, a streamlined classification enhanced analytic clarity and comparability across sites while still allowing us to capture meaningful implementation-relevant patterns.

Although an explicit equity framework was not applied *a priori*, an equity lens was incorporated throughout the analysis, allowing us to identify the need for targeted strategies to expand PrEP access among currently underserved populations. This study did not include the perspectives of individuals who may benefit from PrEP, which represents an important limitation and a critical area for future research—particularly in rural settings. Future studies should include the perspectives of priority populations and employ a priory equity-focused implementation frameworks (or adapt CFIR) and interview guides to assess additional dimensions of PrEP implementation, particularly those related to access, acceptability, and structural inequities.

## Conclusion

These findings advance understanding of PrEP adoption in PHSHCs, highlighting barriers and strategies to expand services. Building on this work, we aim to optimize PrEP implementation across Ontario by fostering a community of practice, supported by expert-led training and telehealth links between sexual health clinics and local providers. Policymakers must urgently address financial and resource barriers limiting access for populations underserved. Sustained reflection, evaluation, and adaptive strategies are essential to ensure equitable, lasting impact. We remain committed to rigorous evaluation to guide evidence-based expansion and establish PrEP as a cornerstone of HIV prevention.

## Data Availability

The datasets presented in this article are not readily available because raw data for this study include transcripts from the qualitative interviews, which may be identifiable if made publicly available. Detailed data summaries are available upon request. Requests to access the datasets should be directed to EN, emma.nagy@southeastph.ca.
