# Supplementary material for: Factors influencing PrEP adoption in sexual health clinics within Ontario’s public health system: a qualitative study using the Consolidated Framework for Implementation Research (CFIR)
Source: Front Public Health. 2026 Apr 22;14:1760989. doi: 10.3389/fpubh.2026.1760989 (PMC13144111; doi:10.3389/fpubh.2026.1760989)
Supplement: Supplementary file 1 [file Supplementary_file_1.docx]

**Interview Guide**

GENERAL INFORMATION OF THE INTERVIEWER AND THE CLINIC

 Could you please start talking a little bit about yourself?

- How long have you worked at the clinic?
- What is your position in the clinic?
- What are your responsibilities and what programs are you part of?
- What is your experience with HIV care and prevention?
- What are your responsibilities and what programs are you part of?

 Could you tell me about the history of this institution?

- (when was it created, its mission, vision, who is part of it?)

Could you give me an example of a recent change in the clinic, be it a protocol for handling an intervention, what have they done? Could you give me an example of how they have done it?

PERCEPTIONS OF PREP

  What do you know about PrEP for HIV prevention?

- knowledge about its effectiveness and the studies on PrEP
- positive and negative beliefs about PrEP

 IMPLEMENTATION OF PrEP IN THE CLINIC

  The clinic has a plan in place to implement PrEP; or do you intend in the future?

- Explore reasons
- Would PrEP be a priority over other programs to be implemented in your clinic?

 What could be a barrier to the implementation of PrEP in the clinic?

- Would PrEP be compatible with the mission, vision of your clinic?
- Does the clinic have the installed capacity (staff, resources, organization, etc.) to implement PrEP?
- Do you think the clinic staff is trained to care for people in PrEP?
- Do you know what the costs in human, financial and infrastructure resources would be for your clinic to implement PrEP?

  What could make it easier for the clinic to implement PrEP in the future?

- Explore personnel, resources, organization, health system …

LEADERSHIP AND INFLUENCES

 Does the clinic work in conjunction with government organizations such as health centers, health posts, etc?

- (Explore with whom and what they do with them)

 Have you discussed the implementation of PrEP with government decision makers at the local or national level? What do you think is the level of government support?

IMPLEMENTATION OF PrEP IN THE CLINIC IN RELATION TO RISK GROUPS

 What are the preventive programs in HIV that are carried out in the clinic?

- (Explore tests, counseling, active case search, etc).
- What works in these programs? and what needs to be improved?
- How has the response and participation of the PHRA been to these programs?
- How has the process of involving PHRA in the clinic's programs? What activities does the institution carry out to facilitate this community participation?

Do you think that PHRA would be willing to use PrEP?

 What risk group do you think should be prioritized to use PrEP? why? Which not? Is there any pressure from PHRA to implement PrEP in your clinic?

TRAINING AND UPDATING OF HEALTH PROVIDERS

 What training has the professionals received in the clinic and how often do they receive training (if they have one)?

- Who is in charge of updating and training staff in the clinic? Are there any incentives or incentives for people in the clinic to be updated?
- If they do not have them, why do not they?
- What have been the themes of the trainings and which have been given the highest priority?
- What do you consider to be the strengths and limitations of the training? What needs improvement in the training programs implemented in the clinic?

 Has your staff received training on PrEP?

- Do you consider it necessary? Why?

  Are you aware of resources, either online or on paper, that are available to help you in implementation of PrEP in your clinic*?  in your practice? (explore the resources)

- What do you think about those resources? have you use any of those resources? (explore usefulness, easiness, simplicity etc)
- what do you think is needed in terms of PrEP education, training or information for you to facilitate the prescription of PrEP or implementation of PrEP in your clinic*?
