# Supplementary material for: Factors influencing PrEP adoption in sexual health clinics within Ontario’s public health system: a qualitative study using the Consolidated Framework for Implementation Research (CFIR)
Source: Front Public Health. 2026 Apr 22;14:1760989. doi: 10.3389/fpubh.2026.1760989 (PMC13144111; doi:10.3389/fpubh.2026.1760989)
Supplement: Supplementary file 2 [file Supplementary_file_2.docx]

**Colour coding examples for CFIR analysis**

| **References** | | **Contrasting narratives** | **Contrasting narratives** | **Contrasting narratives** |
| --- | --- | --- | --- | --- |
|  | **Facilitator** | *When we first launched, we did an actual training session where we just went through kind of step by step how things were going to work in our clinic. The physicians who prescribe PrEP have like a shadow shift with another physician, if needed, before they would do their own clinic.* | *Umm so I I believe it's an important tool in HIV prevention. So I feel positively that it's becoming more readily available to clients.* | *We still are operating our PrEP clinic* |
|  | **Neutral** | *We all have the referral pathway, but it's the actual teaching piece of why it works, how it works, and why it works in a very basic way, yeah.* | *So from what I've learned, prep is very effective if the the largest issue that we see is that. Umm, the barriers in having people to be able to adhere to treatment so I think Prep is very effective.* | *So I don't know that we would have, it would be within our scope unless there was a medical directive put in place. But all those things considered in terms of intention, yes, we would, we would want to if that was a possibility.* |
|  | **Barrier** | *What we're focusing right on right now is building the knowledge and comfort for health, primary care health care providers who are seeing those population to get them comfortable to do it* |  | *Yeah, I think ideally it would be. I think that there are, you know having that situated within primary care is also a huge benefit, right. So the person who's overseeing those individuals for all aspects of their life, it might be better suited there.* |
| BLANK | **Subdomian with no narratives** |  |  |  |

**Characteristics of the intervention across clinics- Colour coding of narratives**

**Outer setting across clinics- Colour coding of narratives**

**Inner setting across clinics- Colour coding of narratives**

**Implementation process across clinics- Colour coding of narratives**
