# Supplementary material for: Factors influencing PrEP adoption in sexual health clinics within Ontario’s public health system: a qualitative study using the Consolidated Framework for Implementation Research (CFIR)
Source: Front Public Health. 2026 Apr 22;14:1760989. doi: 10.3389/fpubh.2026.1760989 (PMC13144111; doi:10.3389/fpubh.2026.1760989)
Supplement: Supplementary file 3 [file Supplementary_file_3.docx]

Barriers and facilitator across clinics for equity adoption of PrEP using the Health Equity Implementation Framework
