# Supplementary material for: Factors influencing PrEP adoption in sexual health clinics within Ontario’s public health system: a qualitative study using the Consolidated Framework for Implementation Research (CFIR)
Source: Front Public Health. 2026 Apr 22;14:1760989. doi: 10.3389/fpubh.2026.1760989 (PMC13144111; doi:10.3389/fpubh.2026.1760989)
Supplement: Supplementary file 4 [file Supplementary_file_4.docx]

**Table 1S. Description of participants from Public Health Sexual Health Clinics**

| **Participant** | **Roles** | **Years of experience in sexual health services** |
| --- | --- | --- |
| PHN#1 | Public Health Nurse, Team Lead | Non available |
| PM#1 | Manager | 21 years |
| PHN#2 | Public Health Nurse Team Lead | 7 years |
| PM#2 | Manager | 8 years |
| PHN#3 | Public Health Nurse | 12 years |
| PM#3 | Manager | 8 years |
| PM#4 | Manager | 20 years |
| PHN#4 | Public Health Nurse | 11 years |
| PHN#5 | Public Health Nurse | 20 years |
| PM#5 | Manager | 6 years |
| PM#6 | Manager | 14 years |
| PHN#6 | Public Health Nurse | 13 years |
| PM#7 | Manager | 2.5 years |
| PM#8 | Registered Practical Nurse, Team Lead | 15 years |
| PM#9 | Manager | 5 years |
| PM#10 | Manager | 2 years |
| PM#11 | Manager | 4 years |
| PHN#7 | Public Health Nurse | More than 20 years of experience |

**Table 2s: Description of clinics using qualitative information- Innovators (Offer PrEP On-Site)**

| **Feature** | **PHSHC#1** | **PHSHC#4** |
| --- | --- | --- |
| Geographic Location | East | Central |
| Recovering from COVID | Yes | Yes |
| Clinic Size | ? | Five nurses on PrEP |
| Physician On-Site | Yes | Yes, five |
| Populations Served | Mainly GBM | Serodiscordant couples, sex workers, GBM |
| Service Modalities | Online, outreach, in person | Online, outreach, in person |
| STI Management | Yes | Yes |
| HIV Case Management | — | Yes |
| HIV Prevention | Yes, testing | Yes |
| Other Services | Birth control | No |
| Networking (Regional) | Yes | No |
| Networking (Universities) | Yes | — |
| Local Networking | Yes (labs) | Yes (labs) |
| NGOs | Yes | Yes |
| Vision & Mission | Yes | Yes |
| Compatibility with Structure | Yes | Yes (adapted) |
| Compatibility with HR | Yes | Yes (adapted) |
| Learning Climate | Like new methods | Like new methods |
| Need Training on PrEP | No | No |
| Time allocated for training | Yes | Yes |
| Resources Available | Yes | Yes |
| Leadership Engagement | Yes | Yes |
| Access to PrEP Knowledge | Yes | Yes |
| Medical Directives | Yes | Yes |

**Table 3s: Description of clinics using qualitative information -Early Adopters**

**(Referral established Path with No On-Site PrEP)**

| **Feature** | **PHSHC#5** | **PHSHC#6** | **PHSHC#9** |
| --- | --- | --- | --- |
| Geographic Location | Central | Central | Southern, mixed rural and urban |
| Recovering from COVID | Yes | Yes | Yes |
| Clinic Size | 10 to 11 nurses | — | Nine nurses, multiple locations |
| Physician On-Site | No | No | No |
| Populations Served | People with STIs, GBM, TGW | GBM, PWUD, youth | Students, underserved areas, immigrants |
| Service Modalities | — | — | In-site, phone/chat service, outreach |
| STI Management | Yes | — | Yes |
| HIV Case Management | Yes | Yes | — |
| HIV Prevention | Yes | — | Yes, rapid testing |
| Other Services | No | Birth control, pregnancy counseling | Harm reduction |
| Networking (Regional) | No | Yes, other PHU | Yes |
| Networking (Universities) | Yes | No | No |
| Local Networking | Yes | Yes | Yes |
| NGOs | Yes | — | Yes |
| Vision & Mission | Holistic vision, social determinants | — | Equity oriented, access |
| Compatibility with Structure | No | Need human resources | No, need lab |
| Compatibility with HR | No, need human resources | Yes | No, need nurse |
| Learning Climate | Motivational interview | No | Yes |
| Need Training on PrEP | Some | No | No |
| Time allocated for training | Yes | Yes | Yes |
| Resources Available | Refer patients, need human resources for prescription | Yes, providers | No, need lab and nurses |
| Leadership Engagement | Yes | Yes | Yes |
| Access to PrEP Knowledge | Yes | Yes | Yes |
| Medical Directives | Yes | — | ? |

**Table 4s: Description of clinics using qualitative information -Early Majority (Rely on external providers)**

|  |  |  |  |  |
| --- | --- | --- | --- | --- |
| **Feature** | **PHSHC#2** | **PHSHC#10** | **PHSHC#8** | **PHSHC#11** |
| Geographic Location | East, rural/urban mix | South Eastern (mainly rural) | Central West | South Eastern |
| Recovering from COVID | Yes | Yes | Yes | No |
| Clinic Size | Five nurses | Geographically large, 6 small clinics | 11 nurses, 2 admin staff | 3 nurses, 1 NP |
| Physician On-Site | No | No | Yes | No |
| Populations Served | GBM, PWUD | GBM, underhoused, PWUD | GBM, underhoused | GBM, underhoused, PWUD |
| Service Modalities | Online, outreach, in-person | Insite, phone/chat/outreach | In-site and outreach | Not specified — |
| STI Management | Yes | Yes | Yes | Yes |
| HIV Case Management | Yes | — | Yes | Yes |
| HIV Prevention | Yes, testing | Yes, testing | Yes, HIV testing | Yes |
| Other Services | Condoms, harm reduction | Harm reduction | Harm reduction, birth control | Harm reduction |
| Networking (Regional) | No | No | Yes, other PHU | No |
| Networking (Universities) | No | No | No | No |
| Local Networking | No | Yes | Yes, primary care doctors | Yes |
| NGOs | Yes | Yes | Yes | Yes |
| Vision & Mission | Decrease HIV infection | Equity-oriented access | Serving underserved populations | Yes |
| Compatibility with Structure | No, need lab | Yes | No | No, there are providers in community |
| Compatibility with HR | No, need resources | No | No, need hire a nurse | No |
| Learning Climate | Yes | Yes | Yes | Yes |
| Need Training on PrEP | Yes | Yes | Yes | Yes |
| Time allocated for training | Yes | Yes | Yes | Yes |
| Resources Available | No | Yes | Need more tools | No |
| Leadership Engagement | No | No | No | No |
| Access to PrEP Knowledge | Yes | Yes | Yes | Yes |
| Medical Directives | Yes | Yes | Yes | No |

**Table 5s: Description of clinics using qualitative information Late Majority,**

**Laggards (Interest or Limited Adoption)**

|  |  |  |  |
| --- | --- | --- | --- |
| **Feature** | **PHSHC#3** | **PHSHC#7** | **PHSHC#12** |
| Geographic Location | North West, mostly rural | South-Western | North West, mostly rural |
| Recovering from COVID | No* | Yes | No |
| Clinic Size | One nurse practitioner, small clinic | Not specified | Not specified |
| Physician On-Site | Yes, but far geographically | No | No |
| Populations Served | Sex workers, Indigenous, underhoused, GBM | GBM, PWUD, youth | Indigenous, GBM, PWUD |
| Service Modalities | Not specified — | Not specified — | Not specified — |
| STI Management | Yes | — | Yes |
| HIV Case Management | No | Yes | Yes |
| HIV Prevention | Yes, HIV testing | Yes | Yes |
| Other Services | — | — | Tuberculosis |
| Networking (Regional) | Yes | — | Yes, but far |
| Networking (Universities) | No | No | No |
| Local Networking | Yes | Yes | No |
| NGOs | Yes | — | Yes |
| Vision & Mission | Felt more primary care | — | Yes |
| Compatibility with Structure | No | No | No |
| Compatibility with HR | No | — | No |
| Learning Climate | Yes | Yes | Yes |
| Need Training on PrEP | Yes | Yes | Yes |
| Time allocated for training | Yes | Yes | Yes |
| Resources Available | No | No | No |
| Leadership Engagement | Yes | Yes | No |
| Access to PrEP Knowledge | Yes | Yes | Yes |
| Medical Directives | Yes | — | No |
